# Effects of Ownership Text Message Wording and Reminders on Receipt of an Influenza Vaccination: A Randomized Clinical Trial

**DOI:** 10.1001/jamanetworkopen.2021.43388

**Published:** 2022-02-17

**Authors:** Alison Buttenheim, Katherine L. Milkman, Angela L. Duckworth, Dena M. Gromet, Mitesh Patel, Gretchen Chapman

**Affiliations:** 1 Department of Family and Community Health, School of Nursing, University of Pennsylvania, Philadelphia; 2 Department of Operations, Information and Decisions, The Wharton School, University of Pennsylvania, Philadelphia; 3 Department of Psychology, University of Pennsylvania, Philadelphia; 4 Behavior Change for Good Initiative, The Wharton School, University of Pennsylvania, Philadelphia; 5 Department of Medicine, Perelman School of Medicine, University of Pennsylvania, Philadelphia; 6 Department of Social and Decision Sciences, Carnegie Mellon University, Pittsburgh, Pennsylvania

## Abstract

**Importance:**

Despite the availability of safe and effective vaccines, many people fail to get vaccinated. Messages using behavioral science principles may increase vaccination rates.

**Objective:**

To determine the effect on influenza vaccination rates of a text message telling patients that an influenza vaccine had been reserved for them.

**Design, Setting, and Participants:**

As part of a larger influenza vaccine messaging megastudy, in this randomized clinical trial, 11 188 patients in 2 large health systems were assigned to receive a text message that stated “a flu shot has been reserved for you,” a text message that stated “flu shots will be available,” or no text message. Both messages included the option to reply yes (Y) or no (N) to indicate that the patient wanted to get vaccinated. Patients 18 years or older were included if they had new or routine (nonsick) primary care appointments scheduled from September 20, 2020, through March 31, 2021.

**Interventions:**

The evening before the scheduled appointment, patients in the 2 message conditions were sent 3 back-to-back SMS messages containing the study wording. Patients in the usual care control group did not receive any study messages.

**Main Outcomes and Measures:**

Receipt of an influenza vaccine on the date of the patient’s scheduled appointment.

**Results:**

A total of 11 188 patients were randomized to the reserved or the available message conditions or to usual care. The 10 158 patients analyzed in the study had a mean (SD) age of 50.61 (16.28) years; 5631 (55.43%) were women; and 7025 (69.16%) were White. According to health records, 4113 (40.49%) had been vaccinated in the previous influenza season, and 5420 (53.36%) were patients at Penn Medicine. In an intent-to-treat analysis, changes in vaccination rates in response to the reserved message did not reach statistical significance (increase of 1.4 percentage points, or 4% [*P* = .31]) compared with the message conveying that influenza vaccines were available. Relative to the usual care control, the reserved message increased vaccination rates by 3.3 percentage points, or 11% (*P* = .004). Patients in the reserved message condition were more likely to text back Y (1063 of 3375 [31.50%]) compared with those in the available message condition (887 of 3351 [26.47%]; χ^2^ = 20.64; *P* < .001), and those who replied Y were more likely to get vaccinated (1532 of 1950 [78.56%]) compared with those who did not (749 of 4776 [15.68%]; χ^2^ = 2400; *P* < .001).

**Conclusions and Relevance:**

This study found that patients who received text messages regarding flu vaccination had greater vaccine uptake than those who received no message. Messages that increase the likelihood that patients will indicate their intention to be vaccinated may also increase vaccination behavior.

**Clinical Trial Registration:**

ClincalTrials.gov Identifier: NCT04565353

## Key Points

**Question:**

Does a text message telling patients that “a flu shot has been reserved for you” increase vaccination rates?

**Findings:**

In a randomized clinical trial of 11 188 patients, a “reserved for you” text message produced vaccination rates that were significantly higher than no message. There was no statistically significant difference in comparison with a message stating that flu shots were available.

**Meaning:**

These findings suggest that text messaging encourages vaccination; more research is needed to evaluate the potential benefits of messages conveying ownership sent before a primary care visit.

## Teaser

This randomized clinical trial assessed whether a text message stating “a flu shot has been reserved for you” increased flu vaccination rates in comparison with a message stating “flu shots will be available.”

## Introduction

The World Health Organization named vaccine hesitancy as one of the top 10 global health threats of 2019.^1^ The COVID-19 pandemic has only increased the importance of encouraging vaccination.^2^ In 2019 to 2020, only 48% of the US adult population received an influenza vaccine,^3^ and national polls indicate that large numbers of Americans remain hesitant to receive one of the COVID-19 vaccines, even as the vaccine is rolled out.^4,5,6,7^

Behavioral interventions can increase vaccine uptake.^8^ For example, automatically scheduling patients for vaccination appointments that they can cancel results in higher vaccination rates than informing patients they can schedule a vaccination appointment.^9^ Furthermore, social processes such as the norm of reciprocity^10^ and mental frames such as scarcity^11^ guide behavior. As part of an influenza vaccine messaging megastudy that tested 19 messages overall,^12^ we crafted a message telling patients that “a flu shot has been reserved for you,” thus framing vaccination as the default or expected action while also harnessing a reciprocity norm and priming a sense of ownership of the vaccine. We hypothesized that this message would increase influenza vaccination rates compared with a similar message that did not use the reserved language and with a usual care control group that received no messages beyond those they would usually receive about an upcoming physician’s appointment.

## Methods

### Study Protocol

The prespecified randomized clinical trial was approved by the institutional review board at the University of Pennsylvania, which granted a waiver of consent for this research. This report follows the Consolidated Standards of Reporting Trials (CONSORT) reporting guideline. The trial protocol and the prespecified statistical analysis plan are included Supplement 1.

**Figure 1.**
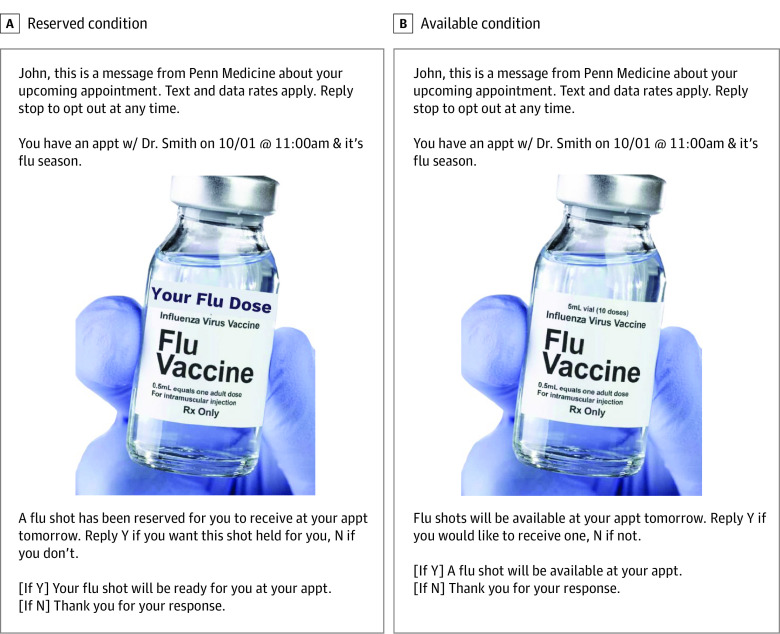
Text Messages Delivered to Patients in 2 Randomized Conditions the Day Before Their Scheduled Office Visit

Our study was conducted within the context of an influenza vaccine messaging megastudy with primary care patients from 2 large health systems (Penn Medicine and Geisinger Health) who had new or routine (nonsick) appointments scheduled from September 20, 2020, to March 31, 2021. Patients were eligible to participate in the study if they (1) had a cell phone number recorded in their electronic health record; (2) had not opted out of receiving SMS appointment reminders from their clinician or asked not to be contacted for research purposes; (3) did not have a documented allergy or adverse reaction to the influenza vaccine; and (4) had not yet received an influenza vaccine in the 2020-2021 flu season according to their electronic health record. In the megastudy, participants were randomized to 1 of 19 messaging groups or a holdout control group as they accrued in the megastudy. In the substudy reported herein (which was designed as a self-contained study within the megatudy), eligible patients were randomized to receive 1 of 2 customized text messages (Figure 1) or usual care. In the 2 message conditions, patients received a sequence of 3 back-to-back SMS messages at 6 pm the evening before their scheduled office visit. The first message provided an appointment reminder; the second message contained a photograph of a vaccine vial with “your flu dose” added to the file in the reserved message condition to further personalize and convey ownership; and the third message conveyed that a shot was “reserved for you” or “available” and requested a response of yes (Y) or no (N). Based on health system records, we tracked influenza vaccine uptake on the patient’s appointment date (even if the original appointment was rescheduled); for usual care patients, we tracked influenza vaccine receipt during the same time window, although no message was sent. If a patient rescheduled on the day of their appointment (at which point they would have already received the study messages) to another date during the study period, we tracked whether they received an influenza vaccine from the date of the original appointment to the date of the rescheduled appointment.

### Statistical Analysis

The primary outcome was receipt of an influenza vaccination on the day of the patient’s appointment, as recorded in the electronic health record, even if the appointment was rescheduled; this was recorded as a binary (0/1) variable. All participants who were randomized to 1 of the 2 message conditions or to the usual care control condition and who met inclusion criteria were included in the intent-to-treat analysis. If a patient canceled their appointment on that day or did not show, it was coded as 0 (unless there was a record of their vaccination on the day of the appointment). We examined the differential effect of the messages on influenza vaccination using linear probability model regressions. Following our preregistered analysis plan, regression analyses predicted receipt of an influenza vaccine during the specified window and included the following covariates: (1) an indicator for being a Penn Medicine patient, (2) patient age (centered), (3) indicators for patient race and ethnicity as captured in the electronic health record, (4) indicators for patient sex, (5) an indicator for whether the patient received an influenza vaccine last year, (6) indicators for the type of clinician who saw the patient, and (7) the linear and squared days (centered) separating the patient’s target primary care appointment from the start of our study (September 20, 2020, when the first patients were enrolled). Race and ethnicity data were collected to assess potential equity implications of the messaging interventions. In coding type of clinicians, the 2 observations with clinician type “other” were combined with the physician assistant category. Analyses were conducted in STATA, version 17.0 (StataCorp LLC). A sample size of 3375 and 3351 patients in the 2 message conditions provided 80% power to detect a 3.2 percentage point difference in influenza vaccine uptake between conditions with 2-sided α = .05 and a baseline vaccination rate of 31%. *P* values were not adjusted for multiple comparisons.

## Results

**Figure 2.**
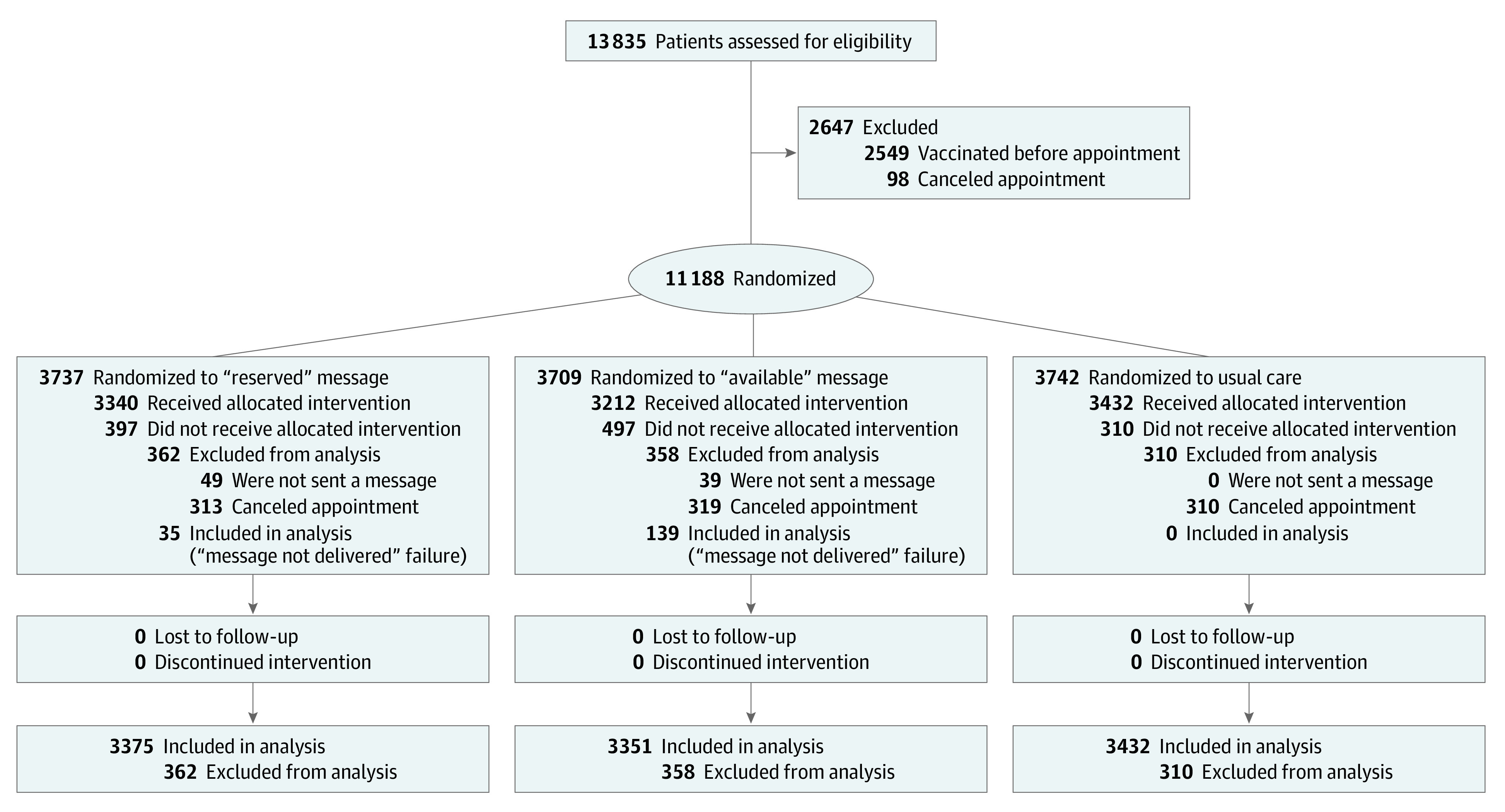
Study Flowchart

A total of 11 188 patients were randomized to receive 1 of 2 customized text messages or usual care (Figure 2). Of the 10 158 patients analyzed, the mean (SD) age was 50.61 (16.28 [range, 18-106]) years; 4527 (44.57%) were men and 5631 (55.43%) were women. With regard to race and ethnicity, 230 patients (2.26%) were Asian; 2058 (20.26%) were Black; 494 (4.86%) were Hispanic; 7025 (69.16%) were White; and 351 (3.46%) were other race or ethnicity (the latter group denotes patients whose race and ethnicity was identified as “other” in the electronic health record). According to health system records, 4113 patients (40.49%) had been vaccinated during the previous influenza season, and 5420 (53.36%) were patients at Penn Medicine (eTable 1 in Supplement 2). In the reserved message condition, 1168 of 3375 patients (34.61%) received an influenza vaccine compared with 1113 of 3351 (33.21%) in the available message condition and 1075 of 3432 patients (31.32%) in the usual care control condition (Table 1). In preregistered regression analyses (Table 2 and eTable 2 in Supplement 2), the 1.4 percentage point (4%) difference between the reserved and available message conditions was not statistically significant, but an exploratory analysis including the usual care control condition demonstrated a significant difference: the vaccination rate in the reserved message condition was 3.3 percentage points (11%) higher than in the usual care control condition, whereas the vaccination rate in the available message condition did not differ from that in the usual care control (eTable 5 in Supplement 2).

**Table 1.** Patients Who Received an Influenza Vaccine in the Present Year According to Study Condition and Patient Characteristics

Characteristic	No./total No. (%) receiving influenza vaccine			
	All conditions	Reserved message	Available message	Usual care control
All	3356/10 158 (33.04)	1168/3375 (34.61)	1113/3351 (33.21)	1075/3432 (31.32)
Appointment month				
September to December 2020	2951/6210 (47.52)	1023/2075 (49.30)	965/2039 (47.33)	963/2096 (45.94)
January to March 2021	405/3948 (10.26)	145/1300 (11.15)	148/1312 (11.28)	112/1336 (8.38)
Race and ethnicity				
Asian	91/230 (39.57)	29/73 (39.73)	32/78 (41.03)	30/79 (37.97)
Black	646/2058 (31.39)	204/672 (30.36)	215/686 (31.34)	227/700 (32.43)
Hispanic	129/494 (26.11)	44/159 (27.67)	41/163 (25.15)	44/172 (25.58)
White	2376/7025 (33.82)	857/2370 (36.16)	784/2299 (34.10)	735/2356 (31.20)
Other^a^	114/351 (32.48)	34/101 (33.66)	41/125 (32.80)	39/125 (31.20)
Influenza vaccine previous year				
Yes	2207/4113 (53.66)	756/1380 (54.78)	743/1359 (54.67)	708/1374 (51.53)
No	1149/6045 (19.01)	412/1995 (20.65)	370/1992 (18.57)	367/2058 (17.83)

^a^ Includes patients whose race and ethnicity was identified as “other” in the electronic health record.

**Table 2.** Coefficients From Preregistered Linear Probability Model Regression Results Comparing Reserved and Available Conditions

Variable	Effect, B coefficient (SE)		
	Model 1: unadjusted (n = 6726)	Model 2: adjusted (n = 6726)	Model 3: with replied Y (n = 6726)
Reserved condition	0.014 (0.012)	0.010 (0.010)	−0.013 (0.009)
Replied Y to text	NA	NA	0.496 (0.010)^a^
Penn Medicine site	NA	0.095 (0.011)^a^	0.023 (0.010)^b^
Age (centered)	NA	0.002 (0.0003)^a^	0.002 (0.000)^a^
Race and ethnicity			
Asian	NA	−0.008 (0.034)	0.026 (0.029)
Black	NA	−0.059 (0.014)^a^	−0.001 (0.012)
Hispanic	NA	−0.033 (0.023)	−0.009 (0.020)
White	NA	1 [Reference]	1 [Reference]
Other^c^	NA	−0.012 (0.028)	0.007 (0.024)
Male sex	NA	0.018 (0.010)^d^	0.016 (0.009)^d^
Clinician			
Physician	NA	0.002 (0.018)	−0.001 (0.015)
Physician assistant	NA	0.003 (0.024)	0.011 (0.020)
Resident	NA	−0.047 (0.027)^d^	−0.050 (0.024)^b^
Nurse practitioner	NA	1 [Reference]	1 [Reference]
Influenza vaccine last year	NA	0.266 (0.010)^a^	0.148 (0.009)^a^
Time since study start (centered)	NA	−0.003 (0.0003)^a^	−0.001 (0.0003)^a^
Time squared (centered)	NA	−0.0000008 (0.000002)	−0.0000003 (0.000002)^d^
Constant	0.332 (0.008)^a^	0.181 (0.020)^a^	0.125 (0.017)^a^
Adjusted *R*^2^	0.0001	0.275	0.462

Abbreviations: NA, not applicable; Y, yes.

^a^ *P* < .001.

^b^ *P* < .05.

^c^ Includes patients whose race and ethnicity was identified as “other” in the electronic health record.

^d^ *P* < .10.

As shown in Table 1, the difference between vaccination rates in the 2 message conditions was directionally higher among patients who had appointments from September to December (1023 of 2075 [49.30%] for reserved and 965 of 2039 [47.33%] for available message conditions), compared with those with appointments from January 2021 to March 2021 (145 of 1300 [11.15%] for reserved and 148 of 1312 [11.28%] for available message conditions). In a preregistered analysis of patients who had appointments from September 2020 to December 2020 (eTable 3 in Supplement 2), the reserved message condition did not have a statistically significant effect on vaccination rates compared with the available message condition (difference of 1.6 percentage points; *P* = .24).

For patients who did not receive an influenza vaccine in the previous year, there was no statistically significant effect of the reserved message condition outperforming the available message condition (χ^2^_1_ = 2.73; *P* = .10 [n = 3987]) (Table 1); among patients who did have a vaccination the previous year, there was no effect on vaccination rates (χ^2^_1_ = 0.003; *P* = .95 [n = 2739]). A preregistered analysis (eTable 4 in Supplement 2) did not detect a significant interaction between the message condition (reserved vs available) and previous influenza vaccine status (B = −0.19 [SE, 0.02]; *P* = .36).

The results presented in Table 1 suggest that the effect of the reserved message occurred only among patients who were not Black. An exploratory analysis (eTable 6 in Supplement 2) revealed that when comparing the vaccination rates observed in the reserved and available message conditions, there was not a significant difference between Black and White patients (message × race interaction, B = −0.29 [SE, 0.025]; *P* = .24). However, when comparing the reserved message condition with the usual care control, the effect of receiving the reserved message was significantly different for Black patients vs White patients (message × race interaction, B = −0.061 [SE, 0.024]; *P* = .01) compared with the usual care control. Receiving the reserved message increased vaccination uptake for White patients (χ^2^_1_ = 13.33; *P* < .001 [n = 4726]) but not Black patients and was directionally negative (χ^2^_1_ = 0.68; *P* = .41 [n = 1372]). Indeed, compared with the usual care control, the 2 message conditions combined had a stronger effect among White patients than among Black patients (F_1, 10140_ = 5.04; *P* = .03) (eTable 6 in Supplement 2).

Patients in the reserved message condition were more likely to text back Y (1063 of 3375 [31.50%]) compared with those in the available message condition (887 of 3351 [26.47%]; χ^2^ = 20.64; *P* < .001), and those who replied Y were more likely to get vaccinated (1532 of 1950 [78.56%]) compared with those who did not (749 of 4776 [15.68%]; χ^2^ = 2400; *P* < .001). As shown in model 3 in Table 2, including the replied variable in the regression numerically reversed the direction of the effect of study condition (B = −0.013 [SE, 0.009]; *P* = .12). A preregistered mediation analysis (eFigure in Supplement 2) revealed an indirect effect (B = 0.032 [95% CI, 0.018-0.045]; *z* = 4.58; *P* < .001), indicating that, compared with the available message, the reserved message was associated with an increased rate of texting back Y, which in turn was associated with a higher vaccination rate. That is, the mechanism through which the reserved message affected vaccination behavior was by increasing the rate of texting back Y.

## Discussion

A text message indicating that “an influenza vaccine has been reserved for you” increased vaccine uptake relative to no message. This study was nested within a larger megastudy that compared 19 message conditions and a no-message usual care control condition^12^; in that study, the 3 message conditions with the highest vaccination uptake were those that used “reserved for you” language, including the reserved message condition in the present study. Whereas the megastudy report includes data reported through December 31, 2020, our present analysis extended to patients with appointments through March 31, 2021. Importantly, whereas the megastudy report compared each message condition with the no-message usual care control condition, we compared the reserved message condition with the available message condition, following our preregistration for this substudy. Patients in both conditions received messages that were similar in all respects with the exception that those in the reserved message condition saw the text “your flu dose” printed on the photograph of the vaccine vial (vs no additional text on the vaccine vial photograph on the available message condition) (Figure 1) and were told that “A flu shot has been reserved for you to receive at your appt tomorrow” rather than “Flu shots will be available at your appt tomorrow.”

The comparison of these 2 messages provides a measure of the effect size of the specific reserved language above and beyond any general effects of receiving a message. This focused comparison reveals a difference between the reserved message and the available message of 1.4 percentage points, which was not statistically significant, with more than 3300 patients per message condition. As in the megastudy report,^12^ we found that vaccination rates were statistically increased in the reserved message condition compared with the no-message usual care control. Our results contribute to the rapidly accumulating evidence on ownership messages to promote vaccination, which have now been tested for COVID-19 vaccination as well as other outcomes. Dai et al^13^ found that an ownership message including language that a COVID-19 vaccine had “just been made available for you” and that patients should “claim their dose” increased COVID-19 vaccination uptake compared with a basic reminder among newly vaccine-eligible patients in January and February 2021.

### Limitations

A limitation of our study is that influenza vaccines received outside of the 2 participating health systems may not have been captured in the electronic health record, which is why our estimates about the effects of messaging on influenza vaccine uptake were limited to vaccines received at the focal medical appointment. We were also unable to observe or influence the patients’ clinical encounters (eg, by observing whether their clinician offered them an influenza vaccine). Another limitation is that our results are generalizable only to patients who are engaged in care (ie, those who have scheduled a medical appointment and have previously consented to receive text messages from their clinician) and may not apply to individuals who are less engaged in care and who do not own a mobile phone or use text messaging. A final limitation is that the study was run during the 2020-2021 influenza season, when patients’ decisions about seeking medical care may have been affected by the COVID-19 pandemic; therefore, our findings may not be generalizable to a more typical influenza season. Our results suggest that text message vaccination reminders are less effective for Black patients than for non-Black patients.

## Conclusions

While this study did not detect a statistically significant difference between the two text message conditions, given the low cost of altering the wording in a text message, it may be useful to consider using a “reserved for you” message when promoting vaccination behavior. However, we caution that variations in message content may sometimes only have incremental benefits for promoting vaccination.

## Notes

Supplement 1.
Trial Protocol

Supplement 2. **eMethods.** Study Participants and Intervention **eResults.** Preregistered and Exploratory Analyses **eFigure.** Mediation Analysis **eTable 1.** Characteristics of Participants in Each Study Condition **eTable 2.** Odds Ratios (SEs) From Preregistered Logistic Regression Results Comparing Reserved and Available Conditions **eTable 3.** Coefficients (SEs) From Preregistered Linear Probability Model Regression for Patients With Appointments Before vs After December 31 **eTable 4.** Coefficients (SEs) From Preregistered Linear Probability Model Regression Results Examining Moderation by Previous Vaccination **eTable 5.** Coefficients (SEs) From Exploratory Linear Probability Model Regression Results Comparing Reserved, Available, and Usual Care Control Conditions **eTable 6.** Coefficients (SEs) From Exploratory Linear Probability Model Regression Results Examining Moderation by Black vs White Race

Supplement 3.
Data Sharing Statement
