# Supplementary material for: Effects of Ownership Text Message Wording and Reminders on Receipt of an Influenza Vaccination: A Randomized Clinical Trial
Source: JAMA Netw Open. 2022 Feb 17;5(2):e2143388. doi: 10.1001/jamanetworkopen.2021.43388 (PMC8855238; doi:10.1001/jamanetworkopen.2021.43388)
Supplement: Supplement 1. — Trial Protocol [file jamanetwopen-e2143388-s001.pdf]

Modification

Basic Info

Confirmation Number:	**deahheae**
Protocol Number:	**843523**
Created By:	**KAY, JOSEPH S**
Principal Investigator:	**MILKMAN, KATHERINE L**
Protocol Title:	**Mega-study to promote flu vaccinations**
Short Title:	**Mega-study to promote flu vaccinations**
Protocol Description:	**We will conduct two "mega-studies" to investigate the effectiveness of at least 40 different SMS and incentive-based interventions for increasing flu vaccinations. Trial A will include primary care patients at Penn Medicine and Geisinger health systems. This project has been approved by Penn Medicine's privacy office, Information systems committee, and Executive Leadership. Trial B will include Walmart pharmacy customers who have consented to receive SMS communications from Walmart.**
Submission Type:	**Social and Biological Sciences**
Application Type:	**EXPEDITED Category 7**

PennERA Protocol Status

Approved (No CR)

Resubmission*
No

Are you submitting a Modification to this protocol?*

Yes

Current Status of Study

Study Status

Closed to subject enrollment (remains active)

If study is currently in progress, please enter the following

Number of subjects enrolled at Penn since the study was initiated

51208

Actual enrollment at participating centers

858489

If study is closed to further enrollment, please enter the following

Number of subjects in therapy or intervention

0

Number of subjects in long-term follow-up only

0

IRB Determination
If the change represents more than minimal risk to subjects, it must be reviewed and approved by the IRB at a convened meeting. For a modification to be considered more than minimal risk, the proposed change would increase the risk of discomfort or decrease benefit. The IRB must review and approve the proposed change at a convened meeting before the change can be implemented unless the change is necessary to eliminate an immediate hazard to the research participants. In the case of a change implemented to eliminate an immediate hazard to participants, the IRB will review the change to determine that it is consistent with ensuring the participant′s continued welfare. Examples: Convened Board Increase in target enrollment for investigator initiated research or potential Phase I research Expanding inclusion or removing exclusion criteria where the new population may be at increased risk Revised risk information with active participants Minor risk revisions that may affect a subject′s willingness to continue to participate Expedited Review Increase in target enrollment at Penn where overall enrollment target is not exceeded or potentially sponsored research Expanding inclusion or removing exclusion where the new population has the same expected risk as the previous, based on similarities of condition Revised risk information with subjects in long-term follow-up Minor risk revisions with no subjects enrolled to date
Expedited Review

Modification Summary
Please describe any required modification to the protocol. If you are using this form to submit an exception or report a deviation, enter 'N/A' in the box below.
This modification adds Sean Ellis, Aden Halpern, and Rayyan Mobarak and removes Tim Lee and Quoc Ho.

Risk / Benefit
Does this amendment alter the Risk/Benefit profile of the study?
No

Change in Consent
Has there been a change in the consent documents?
No

If YES, please choose from the options below regarding re-consenting

Deviations

Are you reporting a deviation to this protocol?*

No

Exceptions

Are you reporting an exception to this protocol?*

No

Protocol Details

Resubmission*
Yes

Hospital Sites
Will any research activities and/or services be conducted at a Penn Medicine affiliated hospital site?
No

Study Personnel

Principal Investigator

Name:	**MILKMAN, KATHERINE L**
Dept / School / Div:	**709 - Operations, Information and Decisions**
Campus Address Mail Code	**6340**
Address:	**HUNTSMAN HALL<br>3730 WALNUT ST**
City State Zip:	**PHILADELPHIA PA 19104-6340**
Phone:	**215-898-5873**
Fax:	**-**
Pager:	
Email:	**kmilkman@wharton.upenn.edu**
HS Training Completed:	**Yes**
Training Expiration Date:	
Name of course completed :	**CITI Protection of Human Subjects Research Training - ORA**
GCP Training Completed:	**No**
Training Expiration Date:	
Name of course completed :	

Study Contacts

Name:	**KAY, JOSEPH S**
Dept / School / Div:	**10732 - Behavior Change for Good**
Campus Address Mail Code	**6241**
Address:	**PSYCHOLOGY<br>3720 WALNUT ST**
City State Zip:	**PHILADELPHIA PA 19104-6241**
Phone:	**-**
Fax:	**-**
Pager:	
Email:	**jskay@wharton.upenn.edu**
HS Training Completed:	**Yes**
Training Expiration Date:	
Name of course completed :	**CITI Protection of Human Subjects Research Training - ORA**
GCP Training Completed:	**No**
Training Expiration Date:	
Name of course completed :	

Name:	**GOODWIN, DENA M**
Dept / School / Div:	**10732 - Behavior Change for Good**
Campus Address Mail Code	**6340**
Address:	**HUNTSMAN HALL<br>3730 WALNUT ST**
City State Zip:	**PHILADELPHIA PA 19104-6340**
Phone:	**215-898-1212**
Fax:	**-**
Pager:	
Email:	**denag@wharton.upenn.edu**
HS Training Completed:	**Yes**
Training Expiration Date:	
Name of course completed :	**CITI Protection of Human Subjects Research Training - ORA**
GCP Training Completed:	**No**
Training Expiration Date:	
Name of course completed :	

Name:	**ELLIS, SEAN F**
Dept / School / Div:	**10732 - Behavior Change for Good**
Campus Address Mail Code	
Address:	
City State Zip:	
Phone:	
Fax:	
Pager:	
Email:	**sfellis@wharton.upenn.edu**
HS Training Completed:	**Yes**
Training Expiration Date:	
Name of course completed :	**CITI Protection of Human Subjects Research Training - ORA**
GCP Training Completed:	**No**
Training Expiration Date:	
Name of course completed :	

Other Investigator

Name:	**PATEL, MITESH S**
Dept / School / Div:	**4239 - DM-General Internal Medicine**
Campus Address Mail Code	**6021**
Address:	**BLOCKLEY HALL**<br>**423 GUARDIAN DR**
City State Zip:	**PHILADELPHIA PA 19104-6021**
Phone:	**215-898-3367**
Fax:	**-**
Pager:	
Email:	**mpatel@mail.med.upenn.edu**
HS Training Completed:	**Yes**
Training Expiration Date:	
Name of course completed :	**CITI Protection of Human Subjects Research Training - ORA**
GCP Training Completed:	**No**
Training Expiration Date:	
Name of course completed :	

Responsible Org (Department/School/Division):

709 - Operations, Information and Decisions

Key Study Personnel

Name:	**MOBARAK, RAYYAN**
Department/School/Division:	**Behavior Change for Good**
HS Training Completed:	**Yes**
Training Expiration Date:	
Name of course completed:	**CITI Protection of Human Subjects Research Training - ORA**
GCP Training Completed:	**No**
Training Expiration Date:	
Name of course completed:	

Name:	**EVANS, CHALANDA**
Department/School/Division:	**Health System**
HS Training Completed:	**Yes**
Training Expiration Date:	
Name of course completed:	**CITI Protection of Human Subjects Research Training - ORA**
GCP Training Completed:	**No**
Training Expiration Date:	
Name of course completed:	

Name:	**GANDHI, LINNEA N**
Department/School/Division:	**Wharton School**
HS Training Completed:	**Yes**
Training Expiration Date:	
Name of course completed:	**CITI Protection of Human Subjects Research Training - ORA**
GCP Training Completed:	**No**
Training Expiration Date:	
Name of course completed:	

Name:	**UNGAR, LYLE H**
Department/School/Division:	**Computer and Information Science**
HS Training Completed:	**Yes**
Training Expiration Date:	
Name of course completed:	**CITI Protection of Human Subjects Research Training - ORA**
GCP Training Completed:	**No**
Training Expiration Date:	
Name of course completed:	

Name:	**BUTTENHEIM, ALISON M**
Department/School/Division:	**Family and Community Health**
HS Training Completed:	**Yes**
Training Expiration Date:	
Name of course completed:	**CITI Protection of Human Subjects Research Training - ORA**
GCP Training Completed:	**No**
Training Expiration Date:	
Name of course completed:	

Name:	**OAKES, ALLISON**
Department/School/Division:	**DM-General Internal Medicine**
HS Training Completed:	**Yes**
Training Expiration Date:	
Name of course completed:	**CITI Protection of Human Subjects Research Training - ORA**
GCP Training Completed:	**No**
Training Expiration Date:	
Name of course completed:	

Name:	**UNGAR, LYLE H**
Department/School/Division:	**Computer and Information Science**
HS Training Completed:	**Yes**
Training Expiration Date:	
Name of course completed:	**CITI Protection of Human Subjects Research Training - ORA**
GCP Training Completed:	**No**
Training Expiration Date:	
Name of course completed:	

Name:	**ROTHSCHILD, JAKE**
Department/School/Division:	**Behavior Change for Good**
HS Training Completed:	**Yes**
Training Expiration Date:	
Name of course completed:	**CITI Protection of Human Subjects Research Training - ORA**
GCP Training Completed:	**No**
Training Expiration Date:	
Name of course completed:	

Name:	**LAMBERTON, CATHERINE M**
Department/School/Division:	**Marketing**
HS Training Completed:	**Yes**
Training Expiration Date:	
Name of course completed:	**CITI Protection of Human Subjects Research Training - ORA**
GCP Training Completed:	**No**
Training Expiration Date:	
Name of course completed:	

Name:	**HIRSCH, ALEX**
Department/School/Division:	**Business Economics and Public Policy**
HS Training Completed:	**Yes**
Training Expiration Date:	
Name of course completed:	**CITI Protection of Human Subjects Research Training - ORA**
GCP Training Completed:	**No**
Training Expiration Date:	
Name of course completed:	

Name:	**GRACI, HEATHER**
Department/School/Division:	**Behavior Change for Good**
HS Training Completed:	**Yes**
Training Expiration Date:	
Name of course completed:	**CITI Protection of Human Subjects Research Training - ORA**
GCP Training Completed:	**No**
Training Expiration Date:	
Name of course completed:	

Name:	**SCHWEITZER, MAURICE**
Department/School/Division:	**Operations, Information and Decisions**
HS Training Completed:	**Yes**
Training Expiration Date:	
Name of course completed:	**CITI Protection of Human Subjects Research Training - ORA**
GCP Training Completed:	**No**
Training Expiration Date:	
Name of course completed:	

Name:	**RARESHIDE, CHARLES A**
Department/School/Division:	**DM-General Internal Medicine**
HS Training Completed:	**Yes**
Training Expiration Date:	
Name of course completed:	**CITI Protection of Human Subjects Research Training - ORA**
GCP Training Completed:	**No**
Training Expiration Date:	
Name of course completed:	

Name:	**HMUROVIC, JILLIAN L**
Department/School/Division:	**Risk Management and Decision Processes Center**
HS Training Completed:	**Yes**
Training Expiration Date:	
Name of course completed:	**CITI Protection of Human Subjects Research Training - ORA**
GCP Training Completed:	**No**
Training Expiration Date:	
Name of course completed:	

Name:	**HALPERN, ADEN**
Department/School/Division:	**Behavior Change for Good**
HS Training Completed:	**Yes**
Training Expiration Date:	
Name of course completed:	**CITI Protection of Human Subjects Research Training - ORA**
GCP Training Completed:	**No**
Training Expiration Date:	
Name of course completed:	

Name:	**YADAV, KULDEEP N**
Department/School/Division:	**SM-DN-Biomedical Graduate Studies**
HS Training Completed:	**Yes**
Training Expiration Date:	
Name of course completed:	**CITI Protection of Human Subjects Research Training - ORA**
GCP Training Completed:	**No**
Training Expiration Date:	
Name of course completed:	

Name:	**DUCKWORTH, ANGELA L**
Department/School/Division:	**Psychology**
HS Training Completed:	**Yes**
Training Expiration Date:	
Name of course completed:	**CITI Protection of Human Subjects Research Training - ORA**
GCP Training Completed:	**No**
Training Expiration Date:	
Name of course completed:	

Disclosure of Significant Financial Interests*
Does any person who is responsible for the design, conduct, or reporting of this research protocol have a **FINANCIAL INTEREST**?
No

Penn Intellectual Property*
To the best of the Principal Investigator's knowledge, does this protocol involve the testing, development or evaluation of a drug, device, product, or other type of intellectual property (IP) that is owned by or assigned to the University of Pennsylvania?
No

Certification
I have reviewed the *Financial Disclosure and Presumptively Prohibited Conflicts for Faculty Participating in Clinical Trials* and the *Financial Disclosure Policy for Research and Sponsored Projects* with all persons who are responsible for the design, conduct, or reporting of this research; and all required Disclosures have been attached to this application.
Yes

Social and Biological Sciences

Study Instruments
Discuss the particulars of the research instruments, questionnaires and other evaluation instruments in detail. Provide validation documentation and or procedures to be used to validate instruments. For well know and generally accepted test instruments the detail here can be brief. More detail may be required for a novel or new instrument. For ethnographic studies identify any study instruments to be used (i.e. for deception studies) and describe in detail where, when and how the study will be conducted and who or what are the subjects of study. Note: For more information on how to conduct ethical and valid ethnographic research, follow the link For oral histories or interviews provide the general framework for questioning and means of data collection. If interviews or groups settings are to be audio taped or video taped describe in detail the conditions under which it will take place. Include a copy of any novel or new test instruments with the IRB submission.
This protocol is for two "mega-studies", in which we will simultaneously test numerous behavioral science strategies to increase flu vaccination rates among Penn Medicine and Geisinger Health primary

care patients (Trial A) and Walmart pharmacy customers (Trial B). Our key dependent variable for both trials is whether participants receive a flu shot. For Trial A, Penn Medicine and Geisinger patients with routine (i.e., non-sick) primary care appointments during the Fall 2020 flu season will be randomly assigned to a treatment condition. Patients will be excluded if they: have opted out of text message appointment reminders, have a documented allergy or adverse event to the flu shot, or have already received their 2020 flu shot (documented in their medical record). Intervention content will be delivered via text message to participants from Penn Medicine and Geisinger Health up to four days prior to their scheduled primary care appointments. For Trial B, Walmart pharmacy customers will be included if they have consented to receive text messages about their prescriptions and have received a flu shot from Walmart in the past. Intervention content will be delivered via text message to Walmart pharmacy customers at the start of the flu season. Between Trial A and Trial B, we plan to include at least 40 different experimental conditions designed by the Behavior Change for Good Scientific Team (including researchers at Penn and other institutions), by Geisinger employees, and by Walmart employees. Interventions may vary the content and timing of text messages (including whether messages are interactive or provide links to surveys and videos). Interventions may also include incentives for behaviors of interest (e.g., receiving the flu shot or completing a survey), the details of which would be described in the text messages themselves. Details of each experimental condition have been included as a separate attachment as a modification to this protocol. Text messages may include first name, appointment date and time, provider name, and practice/pharmacy locations. This is in line with the standard appointment reminders that Penn Medicine patients receive, which includes first name, appointment date and time, and department information. Specifically, the Penn Medicine reminders say "PENNMED: (First Name) has an appt (APPT Date /Time)@(SMS Department Abbreviation). To confirm txt YES. To decline txt NO. Txt Help 4help." Any additional information that we include in text messages to participants beyond that does not include HIPAA information. All messages will be reviewed and approved by our partners before being launched.

Group Modifications
Describe necessary changes that will or have been made to the study instruments for different groups.
The content and timing of text messages and incentives will vary according to which intervention participants are assigned. Specific messages and incentive information has been added as a modification.

Method for Assigning Subjects to Groups
Describe how subjects will be randomized to groups.
In Trial A, patients will be randomly assigned to experimental conditions using stratification that accounts for demographics (e.g., age), and recruitment site. Trial B will also randomly assign participants to experimental conditions, and may also use stratification that accounts for demographics (e.g., age).

Administration of Surveys and/or Process
Describe the approximate time and frequency for administering surveys and/or evaluations. For surveys, questionnaires and evaluations presented to groups and in settings such as high schools, focus group sessions or community treatment centers explain how the process will be administered and who will oversee the process. For instance, discuss the potential issues of having teachers and other school personnel administer instruments to minors who are students especially if the content is sensitive in nature. Describe the procedure for audio and videotaping individual interviews and/or focus groups and the storage of the tapes. For instance, if audio tape recording is to be used in a classroom setting, describe how this will be managed if individuals in the class are not participating in the study. Explain if the research involves the review of records (including public databases or registries) with identifiable private information. If so, describe the type of information gathered from the records and if identifiers will be collected and retained with the data after it is retrieved. Describe the kinds of identifiers to be obtained, (i.e. names, social security numbers) and how long the identifiers will be retained and justification for use.
For Trial A, Penn Medicine and Geisinger Health patients with scheduled primary care appointments during the Fall 2020 flu season who receive SMS reminders about their appointments from their health system will be randomly assigned to receive one of our study's SMS interventions.Text messages will be sent up to four days prior to patients scheduled primary care appointments. These messages will be sent via Penn Medicine's WayToHealth Platform. All messages (including participants' responses to messages) will be stored on the HIPAA-compliant WayToHealth platform. Patient phone numbers from their Penn Medicine and Geisinger records will be shared with WayToHealth to deliver the text

messages. Some messages will be personalized based on patient information (e.g., first name) and Penn Medicine/Geisinger appointment information (e.g., practice name), All identifiable patient information will be removed from the dataset that is shared with researchers for analysis. For Trial B, Walmart pharmacy customers who have consented to receive SMS messages about their prescriptions and have received a flu shot from Walmart in the past will receive messages at the start of the flu season. Messages may be personalized based on customer information (e.g., first name) and/or Walmart pharmacy information (e.g., pharmacy location). These messages will be sent via Walmart pharmacy's text messaging platform. All identifiable customer information will be removed from the dataset that is shared with researchers for analysis.

Data Management

Describe how and who manages confidential data, including how and where it will be stored and analyzed. For instance, describe if paper or electronic report forms will be used, how corrections to the report form will be made, how data will be entered into any database, and the person(s) responsible for creating and maintaining the research database. Describe the use of pseudonyms, code numbers and how listing of such identifiers will be kept separate from the research data.

For Trial A, intervention content will be delivered to participants via text messages from the WayToHealth platform. Penn Medicine and Geisinger will provide the patient data and appointment data needed for WayToHealth to deliver the interventions. This may include a unique identifier, medical record number, first and last name, address, appointment date, appointment time, appointment type, practice and physician information, patient town and zip code, date of birth, gender, phone number(s), documented allergy to the flu shot, and documentation of flu shot receipt for the 2020 flu season. Penn Medicine and Geisinger will also indicate which phone numbers have opted out of receiving text message reminders. WayToHealth will receive these data from Penn Medicine via a secure data feed and from Geisinger via a secure upload. There will be data use agreements in place governing the data sharing with WayToHealth and Wharton as required. The Penn Medicine Academic Computing Services (PMACS) will be the hub for the hardware and database infrastructure that will support the project and is where the WayToHealth web portal is based. The PMACS is a joint effort of the University of Pennsylvania's Abramson Cancer Center, the Cardiovascular Institute, the Department of Pathology, and the Leonard Davis Institute. The PMACS provides a secure computing environment for a large volume of highly sensitive data, including clinical, genetic, socioeconomic, and financial information. Among the IT projects currently managed by PMACS are: (1) the capture and organization of complex, longitudinal clinical data via web and clinical applications portals from cancer patients enrolled in clinical trials; (2) the integration of genetic array databases and clinical data obtained from patients with cardiovascular disease; (3) computational biology and cytometry database management and analyses; (4) economic and health policy research using Medicare claims from over 40 million Medicare beneficiaries. PMACS requires all users of data or applications on PMACS servers to complete a PMACS-hosted cybersecurity awareness course annually, which stresses federal data security policies under data use agreements with the university. After the end of the intervention, patient and intervention data will be transferred from WayToHealth, Penn Medicine, and Geisinger to the Leonard Davis Institute's Health Services Research Data Center (HSRDC), which is maintained at a high security level in accordance with federal regulations (e.g., HIPAA) governing secure computer systems. This will include all data used for study implementation (i.e., the full list included above), as well as additional data to be used in analysis. The additional data may include patient diagnosis, procedures, and prescription information from the previous 5 years, marital status, and whether they have children, race/ethnicity, Charlson Comorbidity score, insurance type, a record of previous flu vaccinations from the previous 5 years, a record of all doctor visits in the past year, appointment arrival time, preferred pharmacy, number of emergency contacts and relation to emergency contacts, patient body mass index, smoking status, record of prior positive test for the flu, and primary care physician (PCP) specialty. Storage and analysis protocols for the data on the LDI HSRDC will be included in the data use agreement with Geisinger and Walmart. All identifiable data will be removed from this list as soon as possible, and will not be used for analysis. The Geisinger investigators will have access to data on (1) any intervention(s) they design, and (2) intervention data for Geisinger patients across all interventions for the purpose of running internal analyses through the HSRDC. They will handle all data in accordance with Geisinger and IRB policies. For Trial B, Walmart pharmacy will deliver the interventions directly to eligible Walmart pharmacy customers via their own text messaging platform. At the end of the intervention, Walmart pharmacy will transfer intervention data (e.g., to which intervention each customer was assigned, text messages sent and received) along with customer pharmacy and medical data (e.g., whether they received a flu shot, previous medical diagnoses) and customer demographic data (e.g., age, gender) to the HSRDC for analysis. Identifiable data will not be

shared. For both Trial A and Trial B, collaborators at Penn and other institutions will access and analyze the non-identifiable data on participants assigned to the interventions they designed through the HSRDC. The server does not allow data to be copied or moved off of the server. Access will only be granted to investigators at other institutions after a data use agreement with Penn has been executed.

Radiation Exposure*
Are research subjects receiving any radiation exposure (e.g. X-rays, CT, Fluoroscopy, DEXA, pQCT, FDG, Tc-99m, etc.) that they would not receive if they were not enrolled in this protocol?
No

Human Source Material*
Does this research include collection or use of human source material (i.e., human blood, blood products, tissues or body fluids)?
No

CACTIS and CT Studies*
Does the research involve Center for Advanced Computed Tomography Imaging Services (CACTIS) and CT studies that research subjects would not receive if they were not part of this protocol?
No

CAMRIS and MRI Studies*
Does the research involve Center for Advanced Magnetic Resonance Imaging and Spectroscopy (CAMRIS) and MRI studies that research subjects would not receive if they were not part of this protocol?
No

Cancer Related research not being conducted by an NCI cooperative group*
Does this protocol involve cancer-related studies in any of the following categories?
No

Medical Information Disclosure*
Does the research proposal involve the use and disclosure of research subject's medical information for research purposes?
Yes

CTRC Resources*
Does the research involve CTRC resources?
No

If the answer is YES, indicate which items is is provided with this submission:

Request for HIPAA Waiver of Authorization

Use of UPHS services*
Does your study require the use of University of Pennsylvania Health System (UPHS) services, tests or procedures*, whether considered routine care or strictly for research purposes?
No

Primary Focus*
Sociobehavioral (i.e. observational or interventional)

Protocol Interventions

	**Sociobehavioral (i.e. cognitive or behavioral therapy)**
	**Drug**
	**Device - therapeutic**
	**Device - diagnostic (assessing a device for sensitivity or specificity in disease diagnosis)**
	**Surgical**
	**Diagnostic test/procedure (research-related diagnostic test or procedure)**
	**Obtaining human tissue for basic research or biospecimen bank**
x	**Survey instrument**
	**None of the above**

The following documents are currently attached to this item:

There are no documents attached for this item.

Sponsors

Business Administrator

Name:	**ERNEST, MELANIE**
Dept / School / Div:	**10732 - Behavior Change for Good**
Phone:	**215-898-0683**
Fax:	
Pager:	
Email:	**mernest@wharton.upenn.edu**

Department budget code

000 - 000 - 0 - 000000 - 0000 - 0000 - 0000

Funding Sponsors

Name:	**NATIONAL BUREAU OF ECONOMIC RESEARCH, INC.**
Type:	**UPENN Other Non-Profit Organizations**

Name:	**NATIONAL INSTITUTES OF HEALTH**
Type:	**UPENN Federal**

Funding sponsors billing address
If you have selected a commercial or industry sponsor, please provide the appropriate address and contact information for the Sponsor for the purposes of billing for IRB review fees (initial review, continuing review and convened modification fees apply here). If the Sponsor is not industry/ commercial, this information is not necessary to provide with your application.

Funding sponsors gift
Is this research being funded by a philanthropic gift?
No

Regulatory Sponsor

IND Sponsor

none

000 - 000 - 0 - 000000 - 0000 - 0000 - 0000

Industry Sponsor

None

Project Funding*
Is this project funded by or associated with a grant or contract?
Pending

Sponsor Funding
Is this study funded by an industry sponsor?
No

Status of contract

The following documents are currently attached to this item:

There are no documents attached for this item.

Multi-Center Research

Penn as lead
1. Is this a multi-center study where Penn is serving as the Lead Site or the Penn PI is serving as the Lead Investigator?
Yes

Management of Information for Multi-Center Research
For Trial A, all intervention procedures will be conducted via Penn Medicine's WayToHealth platform. The Penn team will be responsible for reporting any unexpected problems in the implementation of the interventions. For Trial B, all intervention procedures will be conducted on Walmart's own text messaging platform. The Walmart team will be responsible for reporting any unexpected problems in the implementation of the interventions. For both trials, Penn will be the IRB of record and will inform the Geisinger and Walmart teams of all protocol modifications.

Penn irb of record
2. Is this a multi-center study where the Penn IRB will be asked to serve as the IRB of Record for other external study sites?
Yes

Other Sites

Site:	**Walmart**
Contact:	**Diana Dass**
Pi:	
Mail:	
Phone:	
Email:	**Diana.Dass@walmart.com**

Site:	**Geisinger Health**
Contact:	**Maheen Shermohammed**
Pi:	**Christopher Chabris and Michelle Meyer (Geisinger Co-Investigators)**
Mail:	**100 N. Academy Ave., MC 30-40**
	**Danville, PA 17822-3040**
Phone:	**570-218-5851**
Email:	**mshermohammed@geisinger.edu**

Research will include patients at Geisinger (Trial A) and Walmart pharmacy customers (Trial B). Trial A will be conducted through the WayToHealth platform. The Geisinger team will be responsible for providing patient and other data to WayToHealth to implement the interventions. Data will be transferred directly from Geisinger to WayToHealth for study implementation, and then from Geisinger and WayToHealth to the LDI HSRDC for analysis. Trial B will be conducted via Walmart's own text messaging platform. Interventions will be implemented on the text messaging platform according to specifications provided by the Penn team. Data will be transferred directly from Walmart pharmacy to the LDI HSRDC for analysis. The Penn team will be responsible for ensuring that the interventions are implemented correctly, including using the most current version of the protocol and other materials, updating the protocol and other materials.

Protocol

Abstract

Annually, the influenza (flu) causes millions of illnesses, hundreds of thousands of hospitalizations, and tens of thousands of deaths in the United States (CDC, 2020). In this research project, the Behavior Change for Good Initiative (BCFG), Penn Medicine Nudge Unit (PMNU), and Geisinger Behavioral Insights Team (BIT) will conduct two mega-studies, which will simultaneously test multiple behavioral science strategies to increase flu vaccination rates and apply machine learning to identify which interventions worked best for whom. This research will test at least 40 different interventions, designed by experts on the BCFG, PMNU, and BIT teams. Based on eligible patient appointments from the Fall 2019 flu season, we expected that approximately 80,000 Penn Medicine patients and 75,000 Geisinger patients would be randomly assigned to receive an intervention in Trial A. Based on current COVID-adjusted patient volume projections, we now expect 125,600 patients total for the same time period. Trial B will include approximately 1.2 million Walmart pharmacy customers.

Objectives

Overall objectives

This study aims to 1. Identify which text messaging work best to increase flu vaccination uptake overall among Penn Medicine and Geisinger patients (Trial A) and Walmart pharmacy customers (Trial B) during the Fall 2020 flu season, and 2. Identify heterogeneous treatment effects, determining which messages are most effective at improving the health decisions of different subgroups (e.g., based on age, gender).

Primary outcome variable(s)

Trial A: The primary outcome is whether participants receive a flu vaccination at their primary care appointment. Trial B: The primary outcome is whether participants receive a flu vaccination at any time in the fall of 2020 at a Walmart pharmacy.

Secondary outcome variable(s)

Trial A: A secondary outcome is whether participants receive a flu vaccination after their primary care appointment until March 31, 2021, after receiving the intervention. Trial B: A secondary outcome is

whether participants receive a flu vaccination in the before March 31, 2021.

Background
Annually, the influenza causes millions of illnesses, hundreds of thousands of hospitalizations, and tens of thousands of deaths in the United States (CDC, 2020). The most effective way to combat the flu is with an annual vaccine, which can reduce the risk of contracting the flu by 50% and the risk of hospitalization by 80% (Thompson et al, 2018). The CDC recommends that everyone over 6-months old gets an annual flu vaccination, and estimates that in the 2017-2018 flu season alone (i.e., September 2017-March 2018), the flu vaccine prevented over six million illnesses, almost 100,000 hospitalizations, and almost 6,000 deaths (CDC, 2019). Despite these benefits, each year, less than half of Americans get a flu shot (CDC, 2019), leading to unnecessary illnesses. It is estimated that a 5% increase in vaccination rates in 2017-18 would have led to as many as 25,000 fewer hospitalizations (Hughes et al, 2019).

Study Design

Phase*
Not applicable

Design
Participants will be randomly assigned to the different study conditions. Randomization may be stratified by age, clinical site, primary care provider role, and other factors. Care providers will not be aware of the intervention received by the participant.

Study duration
Trial A: Penn Medicine and Geisinger Health patients with primary care appointments from September 1, 2020 to March 31, 2021 will be included. These participants may receive intervention messages up to four days prior to their scheduled appointments. In some conditions, participants will receive additional communications after their appointment about incentives they earned. Trial B: Walmart pharmacy customers will be assigned to an intervention and may receive intervention messages over the course of 4 days at the start of the flu season. They may also receive additional messages about incentives earned after receiving a flu shot.

Resources necessary for human research protection
Describe research staff and justify that the staff are adequate in number and qualifications to conduct the research. Describe how you will ensure that all staff assisting with the research are adequately informed about the protocol and their research related duties. Please allow adequate time for the researchers to conduct and complete the research. Please confirm that there are adequate facilities for the research.
Penn Medicine, Geisinger, Walmart, and WayToHealth routinely collect and use the data that will be used in this study in the course of their normal business operations. They also routinely send patients text messages and encourage flu vaccinations. Research staff have completed necessary human research protection training.

Characteristics of the Study Population

Target population
Trial A: Adult patients with routine (i.e., non-sick) primary care appointments at Penn Medicine and Geisinger during the 2020-2021 flu season will be included. Trial B: Walmart pharmacy customers who consented to receive text messages and have received a flu vaccine at Walmart in the past will be included.

Subjects enrolled by Penn Researchers
80000

Subjects enrolled by Collaborating Researchers
1275000

Accrual
Penn Medicine, Geisinger Health, and Walmart will provide patient and appointment information to WayToHealth to include patients in the research if they are eligible. The estimated sample (i.e., 80,000 Penn Medicine patients, 75,000 Geisinger patients, and 1 million Walmart pharmacy customers) is based on the number of appointments meeting the eligibility criteria during the 2019-2020 flu season at Penn Medicine and Geisinger and the number of Walmart pharmacy customers who have consented to receive messages about their prescriptions, received a flu shot at Walmart in the past and been selected for the study by Walmart. We will include at least 4,000 participants in each condition, giving us at least 80% power to detect a 3% difference in vaccination rates across conditions.

Key inclusion criteria
Trial A: Penn Medicine and Geisinger patients will be included if they: - Have a cell phone number recorded in a Penn Medicine or Geisinger database - Have a new or routine primary care appointment during the Fall 2020 flu season (not a sick visit) Trial B: Walmart pharmacy customers will be included if they: - Have consented to receive text messages about their Walmart prescriptions - Have received a flu shot from Walmart in the past - Have been selected for study inclusion by Walmart

Key exclusion criteria
Trial A: - Has documentation of allergy or adverse event to influenza vaccination in medical records - Has documentation of already receiving their 2020 influenza vaccination prior to randomization in medical records - Has opted out of receiving text message appointment reminders - Has asked not to be contacted for research purposes - Has an appointment with someone other than their primary care physician - Has an appointment with someone other than a physician, resident, nurse practitioner, or physician assistant. Trial B: None

Vulnerable Populations

	**Children Form**
	**Pregnant women (if the study procedures may affect the condition of the pregnant woman or fetus) Form**
	**Fetuses and/or Neonates Form**
	**Prisoners Form**
	**Other**
x	**None of the above populations are included in the research study**

The following documents are currently attached to this item:

There are no documents attached for this item.

Populations vulnerable to undue influence or coercion
Not applicable

Subject recruitment
Trial A: All eligible Penn Medicine and Geisinger patients with routine primary care appointments from September 1, 2020 to March 31, 2021 will be enrolled and randomly assigned to a study condition. Trial B: All eligible Walmart pharmacy customers will be enrolled and randomly assigned to a study condition.

Will the recruitment plan propose to use any Penn media services (communications, marketing, etc.) for outreach via social media avenues (examples include: Facebook, Twitter, blogging, texting, etc.) or does the study team plan to directly use social media to recruit for the research?
No

The following documents are currently attached to this item:

There are no documents attached for this item.

Subject compensation*
Will subjects be financially compensated for their participation?
Yes

The following documents are currently attached to this item:

There are no documents attached for this item.

If there is subject compensation, provide the schedule for compensation per study visit or session and total amount for entire participation, either as text or separate document

Participants will not be compensated for participating. However, some of the interventions will include incentives for a behavior of interest (e.g., receiving the flu shot, completing a survey). Details about intervention specific-incentives will be included in the study descriptions that will be added to this protocol.

Study Procedures

Suicidal Ideation and Behavior
Does this research qualify as a clinical investigation that will utilize a test article (ie- drug or biological) which may carry a potential for central nervous system (CNS) effect(s)?
No

Procedures
For Trial A, Penn Medicine and Geisinger patients with scheduled primary care appointments during the Fall 2020 flu season who receive SMS reminders of their appointments from their health system will be randomly assigned to receive one of the SMS interventions. For Trial B, all eligible Walmart pharmacy customers will be randomly assigned to receive one of the SMS interventions. In both trials, participants will receive text messages with the content and timing varying based on the intervention design. For Trial A, patient and appointment data (including patient name, telephone number, zip code and town of residence, age, and gender, appointment date, time, and type, practice name and location, information about the primary care physician, and a record of whether they received the flu shot) from Penn Medicine and Geisinger, will be shared with WayToHealth on a regular basis for study implementation (e.g., to randomize participants to condition, deliver intervention content and incentives). After study completion, identifiable information will be removed from the data. WayToHealth will transfer the patient and appointment data, in addition to all intervention data (e.g., text messages sent and responses received), to LDI HSRDC for storage and analysis. Penn Medicine and Geisinger will also transfer additional patient information (e.g., records from previous appointments, comorbidities, additional demographic information) to LDI HSRDC for analysis. For Trial B, interventions will be implemented on Walmart's own text messaging platform. Data will be shared with LDI HSRDC for storage and analysis after the end of the study. This data will include intervention data (e.g., intervention assignment, text messages sent and responses received) and pharmacy information (e.g., flu shot receipt, medical diagnoses) and customer demographic data (e.g., age, gender) from Walmart pharmacy records.

The following documents are currently attached to this item:

There are no documents attached for this item.

Deception
Does your project use deception?
No

International Research
Are you conducting research outside of the United States?
No

Analysis Plan
For both trials, the primary outcome will be whether participants receive an influenza vaccination, as recorded in their medical records or customer data. For Trial A, this will include whether they tell their provider they received a vaccination prior to their appointment but after enrollment in our study, receive a vaccination at their appointment, or receive their vaccination at another appointment or location after enrollment in our study (if documented in their medical record). For Trial B, this will include whether they receive the flu shot at a Walmart pharmacy after enrollment in our study. We will use an ordinary least squares (OLS) regression of a binary vaccination receipt indicator at the individual level, adjusting for individual characteristics (e.g., demographics, comorbidities), and visit characteristics (e.g., doctor information, clinic site; Trial A only).

The following documents are currently attached to this item:

There are no documents attached for this item.

Data confidentiality

	**Paper-based records will be kept in a secure location and only be accessible to personnel involved in the study.**
x	**Computer-based files will only be made available to personnel involved in the study through the use of access privileges and passwords.**
	**Prior to access to any study-related information, personnel will be required to sign statements agreeing to protect the security and confidentiality of identifiable information.**
x	**Wherever feasible, identifiers will be removed from study-related information.**
	**A Certificate of Confidentiality will be obtained, because the research could place the subject at risk of criminal or civil liability or cause damage to the subject's financial standing, employability, or liability.**
	**A waiver of documentation of consent is being requested, because the only link between the subject and the study would be the consent document and the primary risk is a breach of confidentiality. (This is not an option for FDA-regulated research.)**
x	**Precautions are in place to ensure the data is secure by using passwords and encryption, because the research involves web-based surveys.**
	**Audio and/or video recordings will be transcribed and then destroyed to eliminate audible identification of subjects.**

Subject Confidentiality
Wherever feasible, identifiers will be removed from study-related information. Precautions are in place to ensure the data are secure by using passwords and encryption. Identifiable information that is required for implementation (e.g., names, telephone numbers) will be stored on WayToHealth and will only be available in order to ensure that the interventions are being implemented correctly. Study data will be transferred from the WayToHealth platform to the LDI HSRDC for storage and analysis (for Trial A) or directly from Walmart pharmacy to the LDI HSRDC (for Trial B). Both WayToHealth and the HSRDC are HIPAA compliant. All analyses will be conducted on the LDI HSRDC. Access to the LDI HSRDC is limited to LDI-affiliated investigators and access is only granted to researchers with the necessary skills and training to analyze confidential data. Requirements for accessing the LDI HSRDC are here: https://ldi.upenn.edu/ldi-health-services-research-data-center/access. Data use agreements will be executed prior to sharing access with any researchers outside of Penn Medicine. Identifiable data will be removed from the dataset before sharing the data with other researchers.

Sensitive Research Information*
Does this research involve collection of sensitive information about the subjects that should be excluded from the electronic medical record?
No

Subject Privacy
Privacy refers to the person's desire to control access of others to themselves. Privacy concerns people, whereas confidentiality concerns data. Describe the strategies to protect privacy giving consideration to the following: The degree to which privacy can be expected in the proposed research and the safeguards

that will be put into place to respect those boundaries. The methods used to identify and contact potential participants. The settings in which an individual will be interacting with an investigator. The privacy guidelines developed by relevant professions, professional associations and scholarly disciplines (e.g., psychiatry, genetic counseling, oral history, anthropology, psychology).
This research does not differ substantially from actions typically performed by participants' health care providers or pharmacy. Participants will be excluded from the research if they have opted out of receiving reminders for their medical appointments. Interactions with participants will be limited to text messages and content sent via text message (e.g., links to websites sent via text message).

Data Disclosure
Will the data be disclosed to anyone who is not listed under Personnel?
Project collaborators on the BCFG Scientific Team at other institutions will be given access to the data via access to the LDI HSRDC (see subject confidentiality for details). The list of such collaborators will be uploaded as a separate attachment in a protocol modification before any data is shared with them. Collaborators will be required to sign a data use agreement prior to receiving access to the data.

*Data Protection**

x **Name**
x **Street address, city, county, precinct, zip code, and equivalent geocodes**
All elements of dates (except year) for dates directly related to an individual and all ages over 89
x **Telephone and fax number**
Electronic mail addresses
Social security numbers
x **Medical record numbers**
Health plan ID numbers
Account numbers
Certificate/license numbers
Vehicle identifiers and serial numbers, including license plate numbers
Device identifiers/serial numbers
Web addresses (URLs)
Internet IP addresses
Biometric identifiers, incl. finger and voice prints
Full face photographic images and any comparable images
Any other unique identifying number, characteristic, or code
None

Does your research request both a waiver of HIPAA authorization for collection of patient information and involve providing Protected Health Information ("PHI") that is classified as a "limited data set" (city/town/state/zip code, dates except year, ages less than 90 or aggregate report for over 90) to a recipient outside of the University of Pennsylvania covered entity?
Yes

The following documents are currently attached to this item:

There are no documents attached for this item.

Tissue Specimens Obtained as Part of Research*
Are Tissue Specimens being obtained for research?
No

Tissue Specimens - Collected during regular care*
Will tissue specimens be collected during regulator clinical care (for treatment or diagnosis)?
No

Tissue Specimens - otherwise discarded*
Would specimens otherwise be discarded?
No

Tissue Specimens - publicly available*
Will tissue specimens be publicly available?
No

Tissue Specimens - Collected as part of research protocol*
Will tissue specimens be collected as part of the research protocol?
No

Tissue Specimens - Banking of blood, tissue etc. for future use*
Does research involve banking of blood, tissue, etc. for future use?
No

Genetic testing
If genetic testing is involved, describe the nature of the tests, including if the testing is predicative or exploratory in nature. If predictive, please describe plan for disclosing results to subjects and provision of genetic counseling. Describe how subject confidentiality will be protected Note: If no genetic testing is to be obtained, write: "Not applicable."
Not applicable

Consent

1. Consent Process

Overview
We are requesting a waiver of informed consent (see below).

Children and Adolescents
Not applicable

Adult Subjects Not Competent to Give Consent
Not applicable

2. Waiver of Consent

Waiver or Alteration of Informed Consent*
Waiver or alteration of required elements of consent

Minimal Risk*
Participation in this study does not substantially differ from what patients typically experience in the way of appointment-related communications from their health system (Trial A) or customers typically experience when they receive messages from their pharmacy (Trial B). For Trial A, Penn Medicine and Geisinger patients will receive text messages prior to their scheduled appointments about receiving a flu vaccination. As part of their normal operations, Penn Medicine and Geisinger Health send patients appointment reminders prior to their scheduled appointments and encourage their patients to get the flu vaccination. This project has received approval from the Penn Medicine privacy office, Information systems committee, and Penn medicine executive leadership. The study has been socialized with the Geisinger Health Immunization Steering Committee, which has representatives from Marketing, the Care Gaps team, infectious disease, Pharmacy, and the Geisinger Health system CMO, and there were no concerns. For Trial B, Walmart pharmacy customers will be included if they already receive pharmacy text messages from Walmart.

Impact on Subject Rights and Welfare*
Subjects' rights and welfare will not be adversely affected by this waiver. Subjects are free to seek or forego flu vaccination at any time during or after the study, at their appointment, pharmacy, or any other vaccination provider.

Waiver Essential to Research*
It is not feasible to obtain informed consent from subjects in this study. It would be logistically difficult to obtain informed consent from subjects before texting them intervention messages. Furthermore, for many patients or customers, introducing the idea of research into their relationship with their health care provider or pharmacy would be the most negative part of the entire experience in the study. Patients expect to receive communications from their provider about their appointments and from their pharmacy about flu shots but do not expect to be communicating about research. Bringing up research could also cause the surprised patients to refuse -- possibly to the detriment of their health -- flu vaccination options that they would otherwise have accepted.

Additional Information to Subjects
Participants will not receive additional information about the research.

Written Statement of Research*
No

If no written statement will be provided, please provide justificiation

The following documents are currently attached to this item:

There are no documents attached for this item.

Risk / Benefit

Potential Study Risks
This study poses minimal risks to health or well being. Flu shots are sometimes accompanied by side effects, but the subjects in this study would have access to flu shots in the absence of the intervention, and the benefits of becoming inoculated substantially outweigh the risks.

Potential Study Benefits
The current research is aimed at finding better methods for encouraging individuals to receive vaccinations to improve their health. We expect that our results will provide insights that health care providers can use to encourage vaccinations. In addition, participants may be more likely to receive a flu shot, which will make them less susceptible to the flu. See https://www.cdc.gov/flu/prevent/vaccine-benefits.htm for the benefits of the flu shot.

Alternatives to Participation (optional)

Data and Safety Monitoring
The PIs will be responsible for monitoring the study. Data will only be accessible on password- and firewall-protected servers to ensure that only the researchers have access to it.

The following documents are currently attached to this item:

There are no documents attached for this item.

Risk / Benefit Assessment
This study poses minimal risks to health or well being. Flu shots are sometimes accompanied by side effects, but the subjects in this study would have access to flu shots in the absence of the intervention, and the benefits of becoming inoculated substantially outweigh the risks. We believe the potential benefits of this research (e.g., finding a more effective way to encourage people to receive the flu vaccine) considerably outweigh the risks.

General Attachments

The following documents are currently attached to this item:

Cover Letter (coverletter20210621.docx)
