# Supplementary material for: Effects of Ownership Text Message Wording and Reminders on Receipt of an Influenza Vaccination: A Randomized Clinical Trial
Source: JAMA Netw Open. 2022 Feb 17;5(2):e2143388. doi: 10.1001/jamanetworkopen.2021.43388 (PMC8855238; doi:10.1001/jamanetworkopen.2021.43388)
Supplement: Supplement 2. — eMethods. Study Participants and Intervention eResults. Preregistered and Exploratory Analyses eFigure. Mediation Analysis eTable 1. Characteristics of Participants in Each Study Condition eTable 2. Odds Ratios (SEs) From Preregistered Logistic Regression Results Comparing Reserved and Available Conditions eTable 3. Coefficients (SEs) From Preregistered Linear Probability Model Regression for Patients With Appointments Before vs After December 31 eTable 4. Coefficients (SEs) From Preregistered Linear Probability Model Regression Results Examining Moderation by Previous Vaccination eTable 5. Coefficients (SEs) From Exploratory Linear Probability Model Regression Results Comparing Reserved, Available, and Usual Care Control Conditions eTable 6. Coefficients (SEs) From Exploratory Linear Probability Model Regression Results Examining Moderation by Black vs White Race [file jamanetwopen-e2143388-s002.pdf]

Supplementary Online Content

Buttenheim A, Milkman KL, Duckworth AL, Gromet DM, Patel M, Chapman G. Effects of ownership text message wording and reminders on receipt of an influenza vaccination: a randomized clinical trial. *JAMA Netw Open*. 2022;5(2):e2143388. doi:10.1001/jamanetworkopen.2021.43388

eMethods. Study Participants and Intervention
eResults. Preregistered and Exploratory Analyses
eFigure. Mediation Analysis
eTable 1. Characteristics of Participants in Each Study Condition
eTable 2. Odds Ratios (SEs) From Preregistered Logistic Regression Results Comparing Reserved and Available Conditions
eTable 3. Coefficients (SEs) From Preregistered Linear Probability Model Regression for Patients With Appointments Before vs After December 31
eTable 4. Coefficients (SEs) From Preregistered Linear Probability Model Regression Results Examining Moderation by Previous Vaccination
eTable 5. Coefficients (SEs) From Exploratory Linear Probability Model Regression Results Comparing Reserved, Available, and Usual Care Control Conditions
eTable 6. Coefficients (SEs) From Exploratory Linear Probability Model Regression Results Examining Moderation by Black vs White Race

This supplementary material has been provided by the authors to give readers additional information about their work.

© 2022 Buttenheim A et al. *JAMA Network Open*.

eMethods. Study Participants and Intervention

The study was included as part of a large megastudy conducted in fall 2020 by Behavior Change for Good in partnership with Penn Medicine and Geisinger Health (see Milkman et al, 2021). Participants in the megastudy included all patients with new or routine (non-sick) primary care appointments at Penn Medicine between September 24, 2020 and March 31, 2021 and at Geisinger Health between September 28, 2020 and March 31, 2021 who met the following eligibility criteria: (1) they had a cell phone number recorded in their electronic health record, (2) they had not opted out of receiving SMS appointment reminders from their healthcare provider or asked not to be contacted for research purposes, (3) they did not have a documented allergy or adverse reaction to the flu vaccine and (4) they had not yet received an influenza vaccine in the 2020-2021 flu season according to their electronic health record.

A total of 74,811 patients were randomized to one of the 19 experimental conditions or a Usual Care Control condition that did not receive any text-based reminders to get a flu vaccine. Randomization was block-stratified with randomly-selected block sizes of 40, 80, and 120, and took place automatically based on patient lists generated via the electronic health record. All patients received standard appointment reminders (the usual care). Full details of the megastudy are reported in Milkman et al. (2021). The current paper reports results from the two messaging conditions designed by the authors as a well as a comparison with the Usual Care condition.

Patients in the Usual Care Control condition received only the standard appointment SMS reminders from their health system, indicating the date, time, and location of their appointments. Patients in the two messaging conditions received a sequence of three back-to-back SMS messages at 6 pm the day before their appointment, as shown in Figure 1. The first SMS contained general information, the second showed a photo of a flu shot vial that was labeled as “your flu dose” in the Reserved condition, and the third SMS used “reserved” vs. “available” language. The dependent variable was a binary indicator for receipt of a flu shot on the date of scheduled appointment

As shown in Figure 2, not all participants received their allocated messages. Phone carriers blocked some participants in the Available condition from receiving the third SMS that comprised the manipulated portion of the message. Among participants in the Available condition, 539 were sent messages between September 23 and October 11, 2020 worded as “Reply Y if you would like to receive a flu shot at your appt tomorrow, N if not.” For 85 of these patients (16%) the third SMS was not delivered. Because the message failures were limited to the Available question, we altered the wording of the message in an attempt to avoid the carrier filters that blocked the message. On October 12 and 13, 2020, participants in the Available condition received the wording “If you would like to receive a flu shot at your appt tomorrow reply Y, or N if you don’t” but message delivery failed for 12 out of the 95 participants (13%). Beginning October 14, 2020 participants in the Available condition received the wording “Flu shots will be available at your appt tomorrow. Reply Y if you would like to receive one, N if not,” which resulted in very few additional message failures (11, or <1% out of 2686 participants). All told, 108 out of 3320 participants in the Available condition experienced message delivery failure compared to only 7 out of 3347 in the Reserved condition. Some additional participants had message delivery failures for other reasons. All participants who experienced message delivery failure were retained in the analysis to preserve the intent to treat analytic plan and to avoid selection bias that might make the two message conditions non comparable to the no message Usual Care Control condition. A robustness check analysis (not shown) repeated our main regression after excluding the 174 participants who experienced message delivery failures. This analysis showed a 0.8 percentage point difference (p=.42) in vaccination rates between the Reserved and Available conditions.

eResults. Preregistered and Exploratory Analyses

Participant characteristics

Characteristics of participants in each study condition are shown in Table S1. There were no statistically significant differences in key sociodemographic characteristics across the two study arms in the present study or in the Usual Care Control.

Main pre-registered analysis: Logistic model

Pre-registered analysis plans are available at https://aspredicted.org/rf4hm.pdf and https://aspredicted.org/np99d.pdf. Table 2 and eTable 2 show pre-registered linear probability model regressions and logistic regressions including all participants in the Reserved and Available conditions. Vaccination rates do not

© 2022 Buttenheim A et al. *JAMA Network Open*.

differ statistically between the two message conditions, but vaccination rates were higher among patients from Penn Medicine compared to Geisinger Health, higher among older patients, lower among Black patients compared to White patients, and higher for men than for women. In addition, vaccination rates were higher among patients who had received a flu shot the previous year and higher among patients who had appointments earlier in the season.

Additional pre-registered analyses: Appointment and previous vaccination

eTable 3shows additional pre-registered analyses. We analyzed separately patients who had appointments prior to Dec 31, 2020 from those who had appointments on or after Jan 1, 2021. The effect of the Reserved message relative to the Available message was directionally larger for patients with earlier appointments, but even among this group the 1.6 percentage point difference was not statistically significant (p=.24).

The Table 1 percentages suggest that the Reserved message was effective only for patients who did not receive a flu shot last year. The comparison between the Reserved vs. Available conditions for patients who did not receive a flu shot in the previous year showed a marginal effect (p=.099), whereas among patients who did vaccinate the previous year there was no effect (p=.954). A pre-registered analysis shown in eTable 4, however, did not detect a significant interaction between message condition (Reserved vs. Available) and previous flu shot status (p=.355 for the interaction).

Exploratory (post-hoc) analyses: Usual Care Control comparison and race/ethnicity

In exploratory analyses (eTable 5) we compared vaccination uptake in each of the message conditions to the Usual Care Control condition. Compared to the Usual Care Control, the Reserved message increased vaccination by 2.8 percentage points (p=.004), while the Available message increased vaccination by only 1.8 percentage points (p=.069).

An exploratory analysis shown in eTable 6 examined moderation by race. When comparing the Reserved and Available conditions, the message effect for Black patients was not significantly smaller than for White patients (p=.238 for the interaction). However, when comparing the Reserved condition to the Usual Care Control, the message effect was significantly smaller for Black than for White patients (p=.012 for the interaction). Indeed, the combined effect of the Reserved vs. Usual Care and the Available vs. Usual Care interactions was significantly greater than zero, indicating that the benefit of seeing any message relative to no message was larger for White patients than for Black patients. These results suggest that message effectiveness may vary across patient populations.

© 2022 Buttenheim A et al. *JAMA Network Open*.

eFigure. Mediation Analysis

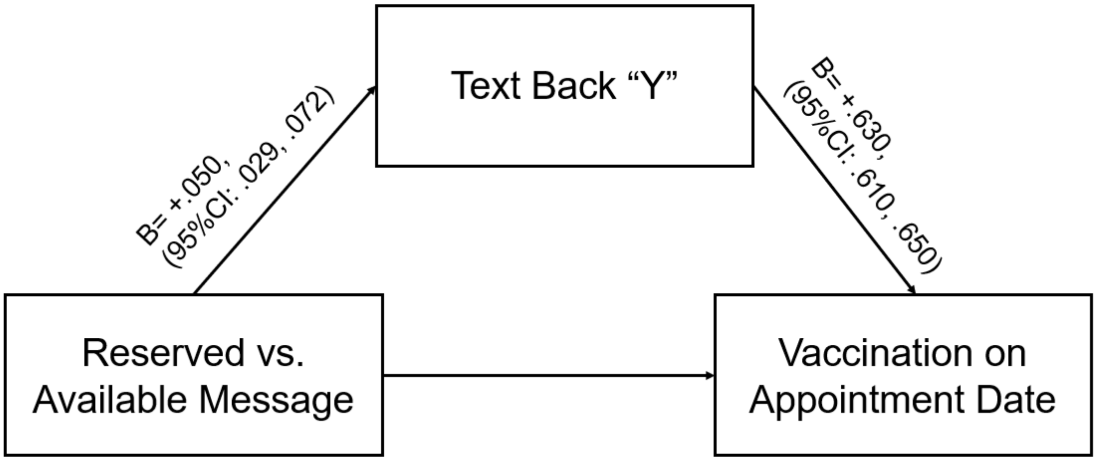

Indirect B= +.032, (95%CI: .018, .045)
Direct B= -0.18, (95%CI: -.036, .000)
Total B= +.014 (95%CI: -.009, .036)

© 2022 Buttenheim A et al. *JAMA Network Open*.

eTable 1. Characteristics of Participants in Each Study Condition

Variable	Condition		
	**Reserved (n=3375)**	**Available (n=3351)**	**Usual Care (n=3432)**
**Site**			
Penn site	53.51%	53.06%	53.50%
Geisinger site	46.49%	46.94%	46.50%
Age (mean)	50.76	50.60	50.48
**Race/Ethnicity**			
Asian	2.16%	2.33%	2.30%
Black	19.91%	20.47%	20.40%
Hispanic	4.71%	4.86%	5.01%
Other	2.99%	3.73%	3.64%
White	70.22%	68.61%	68.65%
**Gender**			
Male	44.21%	44.29%	45.19%
Female	55.79%	55.71%	54.81%
**Provider type**			
Physician	76.36%	75.02%	76.17%
Physician assistant	9.36%	10.21%	9.76%
Resident	4.89%	5.94%	5.07%
Other	0.00%	0.06%	0.00%
Nurse practitioner	9.39%	8.77%	9.00%
**Received influenza vaccine last year**			
Yes	40.89%	40.56%	40.03%
No	59.11%	59.44%	59.97%

Note: Percentages shown are the fraction of participants in a given condition who matched each characteristic)

© 2022 Buttenheim A et al. *JAMA Network Open*.

eTable 2. Odds Ratios (SEs) From Preregistered Logistic Regression Results Comparing Reserved and Available Conditions

Variable	Model 1	Model 2
Reserved condition	1.064 (.055)	1.068 (.065)
Penn site		1.804 (.127)***
Age (centered)		1.010 (.002)***
Asian race/ethnicity		1.006 (.203)
Black race/ethnicity		0.699 (.061)***
Hispanic race/ethnicity		0.780 (.119)
Other race/ethnicity		1.068 (.190)
White race/ethnicity		REF
Male gender		1.110 (.069)*
Physician provider		0.992 (.109)
Physician assistant provider		0.996 (.149)
Resident provider		0.710 (.127)#
Nurse practitioner provider		REF
Flu shot last year		4.257 (.266)***
Time since study start (centered)		0.999 (.002)
Time squared (centered)		1.000 (.000)***
Constant	0.497 (.018)***	0.148 (.019)***
N	6726	6726
Pseudo R^2	.0002	.2500

#p<.10, *p<.05, **p<.01 ***p<.001

© 2022 Buttenheim A et al. *JAMA Network Open*.

eTable 3. Coefficients (SEs) From Preregistered Linear Probability Model Regression for Patients With Appointments Before vs After December 31

**Variable**	**Model 1 Appointment Sep 23-Dec 31**	**Model 2 Appointment Jan1 -Mar 31**
Reserved condition	.016 (.014)	-.001 (.012)
Penn site	.159 (.016)***	-.007 (.014)
Age (centered)	.002 (.000)***	.000 (.0000)
Asian race/ethnicity	-.036 (.046)	.064 (.044)
Black race/ethnicity	-.105 (.020)***	.019 (.017)
Hispanic race/ethnicity	-.055 (.035)	-.008 (.027)
Other race/ethnicity	-.033 (.042)	.068 (.032)*
White race/ethnicity	REF	REF
Male gender	.013 (.014)	.023 (.012)#
Physician provider	.005 (.025)	.-011 (.022)
Physician assistant provider	-.021 (.034)	.019 (.028)
Resident provider	-.093 (.040)*	-.005 (.032)
Nurse practitioner provider	REF	REF
Flu shot last year	.348 (.015)***	.116 (.013)***
Time since study start	.002 (.001)#	-.007 (.003)*
Time squared	-.000 (.000)***	.000 (.000)
Constant	-.012 (.050)	.326 (.075)***
N	4114	2612
Adj R^2	.207	.071

#p<.10, *p<.05, **p<.01 ***p<.001

© 2022 Buttenheim A et al. *JAMA Network Open*.

eTable 4. Coefficients (SEs) From Preregistered Linear Probability Model Regression Results Examining Moderation by Previous Vaccination

**Variable**	**Model 1**
Reserved condition	.018 (.013)
Penn site	0.095 (.011)***
Age (centered)	.002 (.000)***
Asian race/ethnicity	-.008 (0.34)
Black race/ethnicity	-.059 (.014)***
Hispanic race/ethnicity	-.034 (.023)
Other race/ethnicity	.012 (.028)
White race/ethnicity	REF
Male gender	.018 (.010)#
Physician provider	.002 (.018)
Physician assistant provider	.003 (.024)
Resident provider	-.046 (.027)
Nurse practitioner provider	REF
Flu shot last year	.275 (.014)***
Time since study start	-.003 (.000)***
Time squared	-.000 (.000)
Flu shot last year x Reserve	-.019 (.020)
Constant	.177 (.020)***
N	6726
Adj R^2	.275

#p<.10, *p<.05, **p<.01 ***p<.001

© 2022 Buttenheim A et al. *JAMA Network Open*.

eTable 5. Coefficients (SEs) From Exploratory Linear Probability Model Regression Results Comparing Reserved, Available, and Usual Care Control Conditions

Variable	Model 1	Model 2
Reserved condition	.033 (.011)**	.028 (.010)**
Available condition	.019 (.011)#	.018 (.010)#
Penn site		.091 (.009)***
Age (centered)		.002 (.0002)***
Asian race/ethnicity		.013 (.027)
Black race/ethnicity		-.045 (.011)***
Hispanic race/ethnicity		-.026 (.019)
Other race/ethnicity		.006 (.023)
White race/ethnicity		REF
Male gender		.016 (.008)*
Physician provider		.017 (.014)
Physician assistant provider		.013 (.019)
Resident provider		-.002 (.022)*
Nurse practitioner provider		REF
Flu shot last year		.260 (.008)***
Time since study start		-.003 (.0003)***
Time squared		.000 (.000)
Constant	.313 (.008)***	.150 (.017)***
N	10,158	10,158
Adj R^2	.0006	.2748
F-test comparing Reserved to Available	F(1,10155)=1.48, p=.22	F(1,10142)=1.14, p=.29

#p<.10, *p<.05, **p<.01 ***p<.001. Note: The Usual Care Control condition did not have the option to reply (as they did not receive a study message). Consequently the replied variable is not included in these analyses.

© 2022 Buttenheim A et al. *JAMA Network Open*.

eTable 6. Coefficients (SEs) From Exploratory Linear Probability Model Regression Results Examining Moderation by Black vs White Race

**Variable**	**Model 1**<br>**2 conditions**	**Model 2**<br>**3 conditions**
Reserved condition	.016 (.011)	.040 (.011)***
Available condition		.024 (.011)*
Penn site	.095 (.011)***	.090 (.009)***
Age (centered)	.002 (.000)***	.002 (.0002)***
Asian race/ethnicity	-.008 (.034)	.014 (.027)
Black race/ethnicity	-.044 (.019)*	-.014 (.018)
Hispanic race/ethnicity	-.033 (.023)	-.025 (.019)
Other race/ethnicity	.013 (.028)	.007 (.023)
White race/ethnicity	REF	REF
Male gender	.018 (.010)#	.017 (.008)*
Physician provider	.002 (.018)	.017 (.014)
Physician assistant provider	.003 (.024)	.013 (.019)
Resident provider	-.047 (.027)#	-.002 (.022)
Nurse practitioner provider	REF	REF
Flu shot last year	.266 (.010)***	.260 (.008)***
Time since study start	-.003 (.000)***	-.003 (.0003)***
Time squared	-.000 (.000)	.000 (.000)
Black x Reserve	-.029 (.025)	-.061 (.024)*
Black x Available		-.032 (.024)
Constant	.177 (.020)***	.143 (.017)***
N	6726	10,158
Adj R^2	.275	.275
Test that coefficients for the two interactions are jointly > 0		F(1,10140) = 5.04, p=.025

#p<.10, *p<.05, **p<.01 ***p<.001

© 2022 Buttenheim A et al. *JAMA Network Open*.
