# Supplementary material for: Effects of Ownership Text Message Wording and Reminders on Receipt of an Influenza Vaccination: A Randomized Clinical Trial
Source: JAMA Netw Open. 2022 Feb 17;5(2):e2143388. doi: 10.1001/jamanetworkopen.2021.43388 (PMC8855238; doi:10.1001/jamanetworkopen.2021.43388)
Supplement: Supplement 3. — Data Sharing Statement [file jamanetwopen-e2143388-s003.pdf]

Data Sharing Statement

Buttenheim. Effects of Ownership Text Message Wording and Reminders on Receipt of an Influenza Vaccination. *JAMA Netw Open*. Published February 17, 2022. doi:10.1001/jamanetworkopen.2021.43388

Data
Data available: No

Additional Information
Explanation for why data not available: Code and data availability information from the parent study (Milkman et al. 2021 PNAS): The data analyzed in this paper were provided by the Geisinger and Penn Medicine health systems. We do not have legal permission to publicly post individual-level data on vaccinations that we received from our healthcare system partners because our partners consider this to be sensitive health data. Data containing individuallevel health information is typically not made publicly available to protect patient privacy (as even if the data are de-identified, it is still possible to re-identify patients from de-identified data). To be as transparent as possible while protecting patient confidentiality, we have posted aggregated data and all of our analysis scripts, done with R and SAS, at https://bit.ly/2YeummU. Researchers interested in using individual-level data to replicate our results should contact Behavior Change for Good (bcfg@wharton.upenn.edu) and must sign the same data non-disclosure agreement to access the data on a protected medical server.
